# Exploring the interdependence of calcium and chloride activation of O_2_ evolution in photosystem II

**DOI:** 10.1007/s11120-024-01094-6

**Published:** 2024-05-03

**Authors:** Alice Haddy, Shilpa Beravolu, Jeremiah Johnston, Hannah Kern, Monica McDaniel, Brandon Ore, Rachel Reed, Henry Tai

**Affiliations:** Department of Chemistry and Biochemistry, University of North Carolina at Greensboro, Greensboro, NC 27402 USA

**Keywords:** Calcium, Chloride, Electron paramagnetic resonance, Oxygen evolution, Photosystem II, Water oxidation

## Abstract

Calcium and chloride are activators of oxygen evolution in photosystem II (PSII), the light-absorbing water oxidase of higher plants, algae, and cyanobacteria. Calcium is an essential part of the catalytic Mn_4_CaO_5_ cluster that carries out water oxidation and chloride has two nearby binding sites, one of which is associated with a major water channel. The co-activation of oxygen evolution by the two ions is examined in higher plant PSII lacking the extrinsic PsbP and PsbQ subunits using a bisubstrate enzyme kinetics approach. Analysis of three different preparations at pH 6.3 indicates that the Michaelis constant, K_M_, for each ion is less than the dissociation constant, K_S_, and that the affinity of PSII for Ca^2+^ is about ten-fold greater than for Cl^−^, in agreement with previous studies. Results are consistent with a sequential binding model in which either ion can bind first and each promotes the activation by the second ion. At pH 5.5, similar results are found, except with a higher affinity for Cl^−^ and lower affinity for Ca^2+^. Observation of the slow-decaying Tyr Z radical, Y_Z_•, at 77 K and the coupled S_2_Y_Z_• radical at 10 K, which are both associated with Ca^2+^ depletion, shows that Cl^−^ is necessary for their observation. Given the order of electron and proton transfer events, this indicates that chloride is required to reach the S_3_ state preceding Ca^2+^ loss and possibly for stabilization of Y_Z_• after it forms. Interdependence through hydrogen bonding is considered in the context of the water environment that intervenes between Cl^−^ at the Cl−1 site and the Ca^2+^/Tyr Z region.

## Introduction

Photosystem II (PSII), the photosynthetic water oxidase of higher plants, algae, and cyanobacteria, produces molecular oxygen from water using light energy from the sun (Dau et al. 2012; Vinyard and Brudvig 2017; Lubitz et al. 2019; Yano and Yachandra 2014). Water oxidation takes place within the oxygen evolving complex (OEC) of PSII at a Mn_4_CaO_5_ cluster in a catalytic cycle referred to as the S-state or Kok cycle, in which the oxidation state of the cluster, designated S_i_ where i = 0–4, increases in coordination with light absorption at the PSII reaction center, P680. Upon reaching the transient S_4_ state, O_2_ is released and the catalytic site returns to the S_0_ state. Electron transfer to P680 occurs via a redox active tyrosine residue, known as Tyr Z or Y_Z_ (Styring et al. 2012; Mino and Kawamori 2008). Once Tyr Z donates an electron to the reaction center P680, it is quickly reduced by the Mn cluster within milliseconds. A second redox active tyrosine, Tyr D or Y_D_, is also located near the Mn cluster at a position related to Tyr Z by rotation about a C2 axis normal to the membrane plane. Tyr D can also donate electrons to P680, but cannot be reduced by the Mn cluster, resulting in a relatively dark-stable radical state that decays over a period of hours to days.

Chloride and calcium are ionic cofactors long known to be required for oxygen evolution activity. Early studies showed that in higher plants chloride is required for oxygen evolution activity and activates with Michaelis constant, K_M_, in the 1–7 mM range (Kelley and Izawa 1978; Miyao and Murata 1985). Recognition of calcium as an essential cofactor came soon after the introduction of PSII preparation methods (Miqyass et al. 2007; Yocum 2008). Studies of the extrinsic subunits in higher plant PSII revealed their role in regulation of both Ca^2+^ and Cl^−^ function (Bricker and Burnap 2005; Roose et al. 2007). Thus it was observed that removal of the PsbP and PsbQ subunits resulted in a previously unknown requirement for Ca^2+^ and PsbP in particular was identified as moderator of Ca^2+^ access to the OEC (Ghanotakis et al. 1984; Miyao and Murata 1984). Access of Cl^−^ is also regulated by the extrinsic subunits and their removal allows rapid exchange of chloride. Removal of the PsbP and PsbQ subunits facilitates complete Cl^−^ depletion, as observed by oxygen evolution activity, and introduces at least a partial Ca^2+^ dependence of oxygen evolution activity which can be made complete using EDTA or other chelators.

Chloride has been studied extensively to understand the nature of its role in the catalytic cycle (Haddy et al. 2017; Pokhrel et al. 2011; Popelkova and Yocum 2007; Imaizumi and Ifuku 2022). It was shown using flash studies that chloride depletion prevents advancement of the S-state cycle past the S_2_ state (Theg et al. 1984; Itoh et al. 1984), an observation that was complemented by electron paramagnetic resonance (EPR) studies showing that in the absence of chloride the low spin (multiline) state of the S_2_ state cannot form, although the high spin (*g* = 4.1) S_2_ state can form (Damoder et al. 1986; Yachandra et al. 1986; Ono et al. 1987). Direct binding in higher plant PSII was demonstrated using ^36^Cl, revealing a single high affinity Cl^−^ site (Lindberg and Andréasson 1996; Lindberg et al. 1993), and a single site, as represented by bromide ion, was observed about 5 Å from the Mn_4_CaO_5_ cluster using EXAFS (Haumann et al. 2006). X-ray diffraction studies of PSII from thermophilic cyanobacteria revealed that chloride appears at two binding sites near the Mn_4_CaO_5_ cluster (Guskov et al. 2009; Murray et al. 2008; Kawakami et al. 2011), each located about 7 Å away. Both chloride ions are ligated to backbone amide nitrogen atoms, but one (termed Cl−1) is also coordinated to a positively charged lysine residue, which implies that it has a significantly higher binding affinity. The Cl−1 chloride is usually associated with the high affinity activating Cl^−^ ion.

Calcium functions as a part of the Mn_4_CaO_5_ cluster, a role that became clear during early X-ray crystallography studies of PSII (Ferreira et al. 2004). The binding of a single high affinity Ca^2+^ per OEC had been previously identified in higher plant PSII using ^45^Ca (Ädelroth et al. 1995). Studies using higher plant PSII found heterogeneous affinity for Ca^2+^, with K_M_ and K_d_ values reflecting a high affinity site of 10–70 μM and a low affinity site of 0.5–2 mM (van Gorkom and Yocum 2005; Miqyass et al. 2007; Yocum 2008). Ca^2+^ depletion of PSII leads to the formation of a unique EPR signal due to the interaction of the S_2_-state Mn cluster with the Tyr Z radical, or S_2_Y_Z_•, which appears as a broad radical signal at *g* = 2 at liquid helium temperatures. The persistence of the Y_Z_• radical indicates that electron transfer from the Mn cluster to Y_Z_• is blocked in the absence of Ca^2+^. This type of signal was observed first for the S_2_ state (Boussac et al. 1989; Ono and Inoue 1990; Sivaraja et al. 1989), although it was some years before the interacting radical was identified as Tyr Z. Similar signals due to the interaction with other S-states have since been induced by illumination at ultralow temperatures (Styring et al. 2012).

In the experiments presented here, the interdependence of Ca^2+^ and Cl^−^ activation in oxygen evolution is explored using a bisubstrate enzyme kinetics approach combined with EPR spectroscopy of Y_Z_• at 77 K and S_2_Y_Z_• at 10 K. Using higher plant PSII lacking the PsbP and PsbQ subunits, the ion cofactors are treated as substrates to determine kinetic constants, including dissociation constants and bisubstrate Michaelis constants for each ion. Results are consistent with a sequential activation model in which either ion can bind first and activation by the second ion is promoted by binding of the first ion. The slow-decaying Y_Z_• radical and the S_2_Y_Z_• state, both due to Ca^2+^ depletion, are found to require Cl^−^ to be observed. This is related to the requirement for Cl^−^ to reach the S_3_ state preceding Ca^2+^ depletion and suggests that Cl^−^ may stabilize Y_Z_• after its formation.

## Materials and methods

### Preparation of PSII samples

PSII-enriched thylakoid membranes were prepared from fresh market spinach by extraction with Triton X-100 as described previously (Berthold et al. 1981) with modifications (Ford and Evans 1983; Franzén et al. 1985). The final preparation was stored in liquid nitrogen in buffer containing 20 mM MES-NaOH, pH 6.3, 0.4 M sucrose, and 15 mM NaCl.

For enzyme kinetics studies, NaCl-washed PSII lacking the PsbP (23 kDa) and PsbQ (17 kDa) subunits was prepared essentially as described previously (Miyao and Murata 1983). PSII membranes as prepared above were incubated in 20 mM MES-NaOH, pH 6.3, and 0.40 M sucrose (SM) buffer containing 1.0–1.5 M NaCl for 30 min on ice in the dark. After centrifuging for 10 min at 17,400 × g in a Beckman Avanti J-25 high speed centrifuge, the PSII pellet was washed twice by centrifugation in the buffer containing 20 mM MES-NaOH, pH 6.3, and 0.40 M sucrose. For some experiments requiring the removal of Ca^2+^, 0.1 mM EDTA was also included in the wash buffer.

Rates of O_2_ evolution activity were measured at 25 °C using a Clark-type O_2_ electrode (Yellow Springs Instruments, model 5331) in the presence of 1 mM phenyl-*p*-benzoquinone (PPBQ) as electron acceptor, as described previously (Bryson et al. 2005). For some experiments, NaCl-washed PSII was further depleted of Ca^2+^ by illuminating in the presence of 1 mM EDTA. O_2_ evolution assays were carried out in buffer containing 0.40 M sucrose and 20–50 mM MES, pH’d to 6.3 or 5.5 with either Ca(OH)_2_ or NaOH. The indicated concentrations of Cl^−^ and/or Ca^2+^ were achieved by the combined addition of NaCl, CaCl_2_, and Ca(OH)_2_. Rates given represent the average of three or more measurements. Activities were normalized to a control value representing 100% in the presence of sufficient Ca^2+^ and Cl^−^, which was generally 300–500 μmol O_2_ mgChl^−1^ h^−1^, depending on the preparation.

### EPR spectroscopy of tyrosine radicals at 77 K

For electron paramagnetic resonance experiments of the Tyr radicals at 77 K, preparation of NaCl-washed PSII was followed directly by treatment with buffer containing the given amounts of Ca^2+^ and Cl^−^. Incubation of PSII-enriched membranes in 1.3 M NaCl was followed by two washes in SM buffer plus 15 mM NaCl by microcentrifugation at 15 k rpm for 5 min; these mild centrifugation conditions helped preserve the integrity of the PSII centers. Separate samples were then washed twice to suspend in SM buffer containing either: 5 mM Ca^2+^ and 25 mM Cl^−^; 25 mM Cl^−^; 5 mM Ca^2+^; or neither Ca^2+^ nor Cl^−^ (achieved by the combined addition of NaCl, CaCl_2_, and Ca(OH)_2_). The PSII concentration was adjusted to 3 mgChl mL^−1^ and samples were incubated in their buffers in the dark for 30 min, with the addition of 1 mM EDTA for samples without Ca^2+^. PPBQ was added to a concentration of 2 mM (from a 50 mM stock solution in dimethylsulfoxide). Samples were transferred to clear-fused quartz EPR tubes (4 mm outer diameter) and frozen in liquid nitrogen.

EPR spectroscopy was carried out using a Bruker Instruments 10/12 EMX model EPR spectrometer. Spectra of the tyrosine radicals were collected using a microwave frequency of 9.45 GHz, microwave power of 1.0 mW, modulation frequency of 100 kHz, and modulation amplitude of 3 G. A liquid nitrogen finger dewar was used to maintain the temperature at 77 K. Illumination was carried out using a 300 W halogen lamp with the light beam directed through a 5 mM CuSO_4_ solution. Samples were prepared in the S_1_ state by illuminating at room temperature for 5 s, then incubating on ice for about 60 min before freezing in liquid nitrogen. The EPR spectrum was taken of the dark-adapted state, which represented reduced Tyr D (Y_D_•) only. Samples were then illuminated in an ice water bath (0 °C) for 30 s and transferred quickly to a dry ice/ethanol bath before cooling completely in liquid nitrogen. A second EPR spectrum was taken after illumination, which represented both Y_D_• and reduced Tyr Z (Y_Z_•). Signals were quantified as the peak-to-trough heights. The amount of Y_Z_• was calculated by comparison with Y_D_•, since they are present in PSII in a 1:1 ratio. A small amount of a single-line radical was also induced by illumination and its contribution to signal heights was estimated in two ways. In the first, an adjustable amount of a model radical signal was subtracted from the light-minus-dark difference spectrum to obtain a “pure” tyrosine radical signal. In the second case, an adjustable amount of the spectrum of the dark-adapted sample was subtracted from that of the illuminated sample until a “pure” simple radical signal was obtained. These two correction methods consistently gave the same amount of Y_Z_• within about 2%.

### EPR spectroscopy of OEC signals at 10 K

For liquid helium EPR experiments of the S_2_-state multiline, dark-stable multiline, and S_2_Y_Z_• signals, NaCl-washed PSII was suspended in SM buffer containing 2 mM EDTA and incubated in the dark on ice for 30 min. After pelleting by high-speed centrifugation, separate samples were prepared in SM buffer containing either: 6 mM Ca^2+^ and 25 mM Cl^−^; 25 mM Cl ^−^; 6 mM Ca^2+^; or neither Ca^2+^ nor Cl^−^. All buffers also contained 0.1 mM EDTA. Samples were centrifuged to pellet and resuspended in the same buffers to a concentration of 10 mgChl mL^−1^. After the addition of PPBQ to a concentration of 2 mM, PSII samples were transferred to EPR tubes and dark-adapted on ice for 80 min before freezing in liquid nitrogen.

EPR spectroscopy was carried out as above, except that an Oxford Instruments ESR 900 liquid He cryostat was used to control the temperature at 10 K. EPR settings included a microwave frequency of 9.65 GHz, microwave power of 20 mW, modulation frequency of 100 kHz, and modulation amplitude of 18 G. For production of the signals, EPR samples were thawed quickly in room temperature water, then illuminated for 15 s at room temperature, followed by dark-adaptation on ice for 1 h. Samples were frozen in liquid nitrogen and the spectrum of the dark-adapted state was taken; under these conditions, the dark-stable multiline signal appears in Ca^2+^-depleted samples. Next samples were illuminated at 195 K (ethanol/dry ice bath) for 4 min, which produces the normal S_2_ state in active samples. Samples were then brought to 273 K (water ice bath) and illuminated for 30 s, which produces the S_2_Y_Z_• signal if electron transfer from the Mn cluster to Tyr Z is inhibited.

### Single substrate and bisubstrate enzyme kinetics analyses

Apparent Michaelis constants, K_M, app_, and apparent maximum velocities, V_max, app_, were determined for individual curves of reaction velocity, v, versus activator (Ca^2+^ or Cl^−^) concentration, treating each as a substrate, S. In most cases, the data were fitted to the Michaelis–Menten equation with the addition of a constant velocity factor, V_0_, to account for the initial activity (Eq. 1). In some cases, the activity decreased with high concentrations of the activator, indicating inhibition due to secondary binding. For these cases, the data were fitted to a modified Michaelis–Menten equation that included a substrate inhibition term, with apparent inhibition constant, K_I, app_, representing the dissociation constant of inhibitory substrate (Eq. 2).

1 $$v=\frac{{V}_{max, app}[S]}{\left[S\right]+{K}_{M,app}}+{V}_{0}$$

2 $$v=\frac{{V}_{max, app}[S]}{\left[S\right]+{K}_{M,app}+{[S]}^{2}/{K}_{I,app}}+{V}_{0}$$

**Scheme 1:**
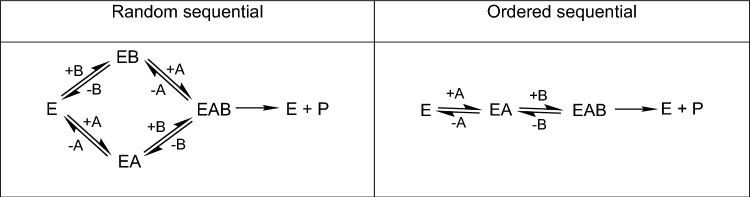
Bisubstrate binding models for enzyme kinetics analyses

To study the interdependence of calcium and chloride in activation of O_2_ evolution, experiments were carried out in which the activation by Cl^−^ was observed in the presence of various concentrations of Ca^2+^, and vice versa. These data were analyzed in terms of sequential binding of the substrate activators, with extreme cases represented by the random sequential (rapid equilibrium) binding model and the ordered sequential binding model, shown in Scheme 1 (Bisswanger 2017; Marangoni 2003; Cornish-Bowden 1995).

For the random sequential model of substrate binding, the reaction velocity v can be related to the concentrations of substrates A and B and overall reaction velocity, V_max_, as given below:

3 $$\frac{1}{v}=\frac{1}{[A]}\left(\frac{{K}_{S}^{A}{K}_{M}^{B}}{{V}_{max}{K}_{S}^{B}}+\frac{{K}_{S}^{A}{K}_{M}^{B}}{{V}_{max}[B]}\right)+\left(\frac{1}{{V}_{max}}+\frac{{K}_{M}^{B}}{{V}_{max}[B]}\right)$$

Here K_S_^A^ and K_S_^B^ are dissociation constants for the binding equilibrium of the first ligand and K_M_^A^ and K_M_^B^ are similar to Michaelis constants, where K_M_^A^ is associated with the binding of A to EB and K_M_^B^ is associated with the binding of B to EA. In this model, all four constants can be found since K_S_^A^K_M_^B^ = K_S_^B^K_M_^A^. The rate equation can be written in alternative forms using this relationship, including that given below:

4 $$\frac{1}{v}=\frac{1}{[A]}\left(\frac{{K}_{M}^{A}}{{V}_{max}}+\frac{{K}_{S}^{A}{K}_{M}^{B}}{{V}_{max}[B]}\right)+\left(\frac{1}{{V}_{max}}+\frac{{K}_{M}^{B}}{{V}_{max}[B]}\right)$$

This version is often favored for the ordered sequential model since the dissociation constant in which the second ligand would bind first, K_S_^B^, is not relevant. For a strictly ordered sequential binding model, only K_S_^A^ and K_M_^B^ are relevant and terms involving K_S_^B^ or K_M_^A^ are omitted from the analysis. However, it is possible to have situations that lie somewhere between the extremes of completely random and strictly ordered sequential binding.

For either Eq. 3 or 4, if a plot of 1/v versus 1/[A] is constructed according to the Lineweaver–Burk method, the slopes and intercepts are related to the second variable substrate concentration [B] and the K_S_ and K_M_ values for each substrate. For the random sequential model, Eq. 3 leads to Lineweaver–Burk slopes and intercepts given by:

5 $$LB\ slope=\frac{{K}_{M, app}^{A}}{{V}_{max, app}}=\frac{{K}_{S}^{A}{K}_{M}^{B}}{{V}_{max}{K}_{S}^{B}}+\frac{{K}_{S}^{A}{K}_{M}^{B}}{{V}_{max}[B]}$$

6 $$LB\ intercept=\frac{1}{{V}_{max, app}}=\frac{1}{{V}_{max}}+\frac{{K}_{M}^{B}}{{V}_{max}[B]}$$

For the Ca^2+^ and Cl^−^ bisubstrate activation data collected in this study, the apparent V_max,app_ and K_M,app_ values determined from individual activation curves of v versus [A] were plotted accordingly against 1/[B] to obtain the K_S_ and K_M_ values for each substrate. In the analysis of these secondary plots, the fits included a weight of the reciprocal variable to account for the error when taking 1/[B] as the x-axis.

## Results

### *Ca*^*2*+^*activation at various Cl*^*−*^* concentrations*

**Fig. 1:**
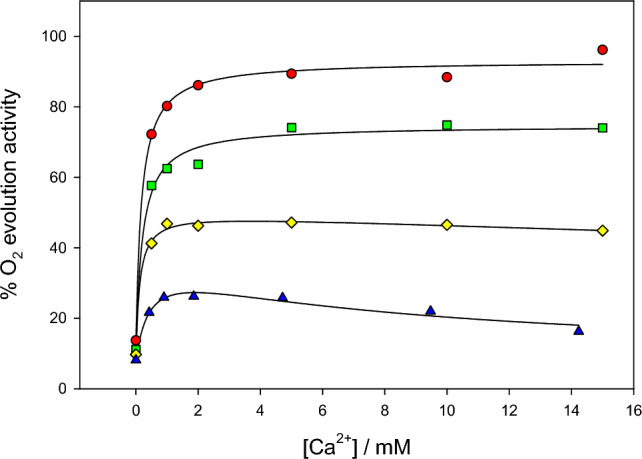
Dependence of O_2_ evolution activity on Ca^2+^ in the presence of various concentrations of Cl^−^ at pH 6.3: red circles, 12.0 mM Cl^−^; green squares, 5.0 mM Cl^−^; yellow diamonds, 2.0 mM Cl^−^; and blue triangles, 1.0 mM Cl^−^. Solid lines show the fits to the data sets. PSII lacking PsbP/PsbQ was depleted of Ca^2+^ as described. Assays were carried out in the presence of 1 mM EDTA; the Ca^2+^ concentrations given were corrected for that complexed with EDTA

The dependence of oxygen evolution activity on Ca^2+^ concentration was studied at various Cl^−^ concentrations at pH 6.3 using NaCl-washed PSII which lacked the PsbP and PsbQ subunits (Fig. 1). Absence of the two subunits facilitates the removal of Cl^−^ and Ca^2+^ ions associated with activation. In the absence of PsbP and PsbQ, the chloride responsible for activation is relatively easy to remove by washing with buffer that does not contain Cl^−^. Removal of activating calcium is more difficult because of the high affinity of PSII for Ca^2+^ and the trace amounts present in solution in the absence of added Ca^2+^. To improve Ca^2+^ depletion, a treatment method was employed in which the PSII was illuminated in the presence of EDTA, as described in Materials and Methods.

The Ca^2+^ activation curves, shown in Fig. 1, were fitted to the Michaelis–Menten equation (Eq. 1) as described in Materials and Methods to obtain apparent Michaelis constants, K_M, app_, and apparent maximum velocities, V_max, app_, for the individual curves. For the lowest Cl^−^ concentrations, the activity decreased slightly with increasing Ca^2+^ concentrations, suggesting suppression due to secondary binding of Ca^2+^. For these cases, the data were fitted to the modified Michaelis–Menten equation that included a substrate inhibition term with apparent inhibition constant K_I, app_ (Eq. 2). In each case the fits also included a V_0_ factor to account for the initial activity, which ranged from 8 to 14% for these samples. When the data were plotted as 1/(v − V_0_) versus 1/[Ca^2+^] according to the Lineweaver–Burk method (not shown), the plots were essentially linear except for deviation caused by the substrate inhibition, consistent with activation from a single type of calcium site.

The values found for K_M, app_(Ca^2+^) from the individual curves ranged from 0.13 to 0.45 mM and showed no apparent trend with Cl^−^ concentration (Table 1). The V_max, app_ values increased with the Cl^−^ concentration, as expected if Cl^−^ is a limiting substrate. At 1 mM Cl^−^, the substrate inhibition effect was characterized by an inhibition dissociation constant, K_I, app_(Ca^2+^), of 8 mM; at 2 mM Cl^−^, this effect was weakened such that K_I, app_(Ca^2+^) was found to be about 106 mM.

**Table 1:** Apparent kinetic constants for Ca^2+^ activation of PSII lacking PsbP/PsbQ at various Cl^−^ concentrations for the activation curves shown in Fig. 1. Values are from direct fits to the Michaelis–Menten equation with substrate inhibition term, where relevant, and corrected for initial activity V_0_. Errors given are standard errors of the fitted values

[Cl^−^] / mM	K_M, app_(Ca^2+^) / mM	V_max, app_	K_I, app_(Ca^2+^) / mM
1.0	0.45 ± 0.24	29 ± 5%	8.0 ± 1.5
2.0	0.13 ± 0.04	40 ± 2%	106 ± 46
5.0	0.22 ± 0.05	63 ± 3%	na
12.0	0.19 ± 0.04	79 ± 3%	na

**Fig. 2:**
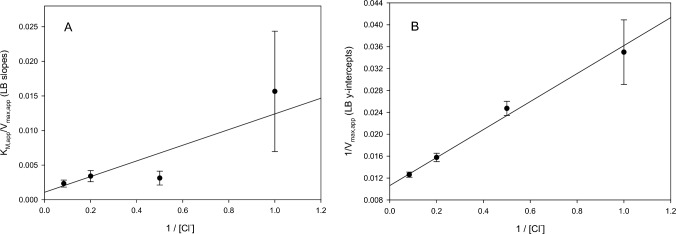
Secondary plots of Cl^−^ dependence of Ca^2+^-activated O_2_ evolution in PSII lacking PsbP/PsbQ: A, Lineweaver–Burk slopes vs. 1/[Cl^−^]; B, Lineweaver–Burk intercepts vs. 1/﻿[Cl^−^]. Data points correspond to the fits shown in Fig. 1, except with the omission of the inhibitory effect observed at high Cl^−^ concentrations as described in the text. Error bars were propagated from the data in Table 1

To obtain overall kinetic constants, the data were analyzed using the sequential binding model of bisubstrate activation as described in Materials and Methods. The apparent kinetic constants found from the direct fits were used to construct secondary plots of K_M,app_(Ca^2+^)/V_max,app_ (equivalent to Lineweaver–Burk slopes) and 1/V_max,app_ (equivalent to Lineweaver–Burk intercepts) versus 1/[Cl^−^] (Fig. 2). By using the apparent K_M,app_ and V_max,app_ values, inaccuracies from the inhibitory effect represented by K_I,app_ and the initial background activity represented by V_0_ were eliminated. From the secondary plots, the constants for Cl^−^ activation, K_S_(Cl^−^) and K_M_(Cl^−^), were found to be 11 ± 13 mM and 2.4 ± 0.2 mM, respectively. The constants for Ca^2+^ activation, K_S_(Ca^2+^) and K_M_(Ca^2+^), were found to be 0.44 ± 0.17 mM and 0.10 ± 0.12 mM, respectively. These are listed in Table 4 along with similar constants found from the additional analyses described in the following sections.

### *Cl*^*−*^* activation at various Ca*^*2*+^*concentrations*

**Fig. 3:**
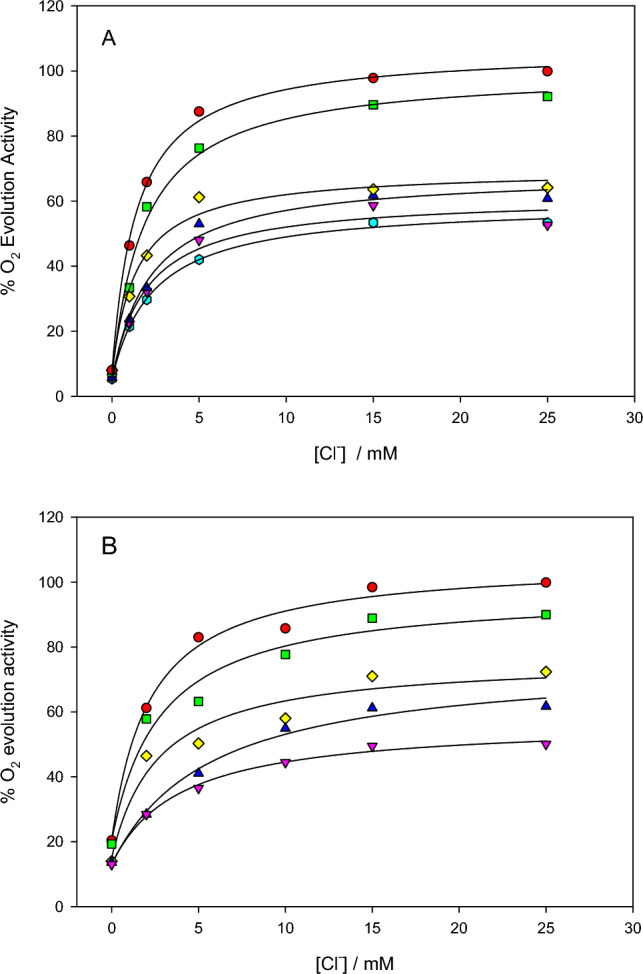
Dependence of O_2_ evolution activity on Cl^−^ concentration in the presence of various concentrations of Ca^2+^ at pH 6.3 using PSII lacking PsbP/PsbQ with: A, no further treatment; B, with Ca^2+^ depletion treatment. Ca^2+^ concentrations were: red circles, 5.0 mM Ca^2+^; green squares, 3.0 mM Ca^2+^; yellow diamonds, 1.0 mM Ca^2+^; blue triangles, 0.50 mM Ca^2+^; pink inverted triangles, 0.25 mM Ca^2+^; and cyan hexagons, 0.10 mM Ca^2+^. Solid lines show direct fits to the data sets

The activation of oxygen evolution by Cl^−^ was studied at various Ca^2+^ concentrations at pH 6.3 using PSII lacking the PsbP and PsbQ subunits (NaCl-washed PSII). In one set of experiments the sample was used without further treatment, while in a second set of experiments the sample was treated with EDTA to remove Ca^2+^, using the same method described in the last section for Ca^2+^ activation experiments. Activation curves are shown in Fig. 3 for Ca^2+^ concentrations ranging from 0.10 to 5.0 mM.

Each individual curve was fitted with the Michaelis–Menten equation including V_0_ term (Eq. 1) to obtain values of V_max,app_ and K_M,app_(Cl^−^). For these data sets, V_0_ ranged from 5 to 20% of the maximum rates. Values found for K_M,app_(Cl^−^) were in the range of 1 to 2.5 mM for PSII lacking PsbP/PsbQ (with no further treatment) and 2 to 6 mM for PSII lacking PsbP/PsbQ with additional Ca^2+^ depletion (Table 2). In each case, there appeared to be a trend in which K_M,app_(Cl^−^) decreased with increasing Ca^2+^ concentration. When viewed using the Lineweaver–Burk method by plotting 1/(v − V_0_) versus 1/[Cl^−^] (not shown), the plots were linear except for deviation caused by substrate inhibition at high [Cl^−^], which is consistent with activation from a single type of chloride site.

**Table 2:** Apparent kinetic constants for Cl^−^ activation of PSII lacking PsbP/PsbQ at various Ca^2+^ concentrations for the activation curves shown in Fig. 3. Values are from direct fits to the Michaelis–Menten equation with V_0_ term. Errors given are standard errors of the fitted values

No further treatment		With Ca^2+^ depletion step	
[Ca^2+^]/mM	K_M,app_(Cl^−^)/mM	[Ca^2+^]/mM	K_M,app_(Cl^−^)/mM
0.10	2.4 ± 0.2	–	–
0.25	2.0 ± 0.7	0.25	4.0 ± 0.7
0.50	2.2 ± 0.5	0.50	5.7 ± 1.5
1.0	1.4 ± 0.4	1.0	2.7 ± 1.3
3.0	1.9 ± 0.4	3.0	2.8 ± 1.1
5.0	1.4 ± 0.1	5.0	2.2 ± 0.5

The activation data for Cl^−^ at various Ca^2+^ concentrations were analyzed in terms of the sequential bisubstrate activation model, as described in Materials and Methods. In this case, the Cl^−^ was assigned as substrate A and Ca^2+^ as substrate B. (Note that the assignment of A and B for analysis purposes has no effect on the values obtained.) Using the fitted values of K_M,app_(Cl^−^) and V_max, app_, secondary plots of the data were made of the K_M,app_(Cl^−^)/V_max,app_ versus 1/[Ca^2+^] and 1/V_max,app_ versus 1/[Ca^2+^]. The bisubstrate kinetic constants found for PSII lacking PsbP/PsbQ without further treatment were: K_S_(Cl^−^) = 2.8 ± 1.6 mM, K_M_(Cl^−^) = 1.6 ± 0.2 mM, K_S_(Ca^2+^) = 0.26 ± 0.11 mM, and K_M_(Ca^2+^) = 0.14 ± 0.06 mM. For PSII lacking PsbP/PsbQ with Ca^2+^ depletion step, the constants were: K_S_(Cl^−^) = 7.5 ± 2.5 mM, K_M_(Cl^−^) = 2.1 ± 0.5 mM, K_S_(Ca^2+^) = 0.90 ± 0.30 mM, and K_M_(Ca^2+^) = 0.25 ± 0.05 mM. In general, K_S_ and K_M_ values found for the preparation with Ca^2+^ depletion treatment were higher than those for the preparation without further treatment. Values are also given in Table 4.

### *Cl*^*−*^* activation at pH 5.5 for varying Ca*^*2*+^*concentrations*

**Fig. 4:**
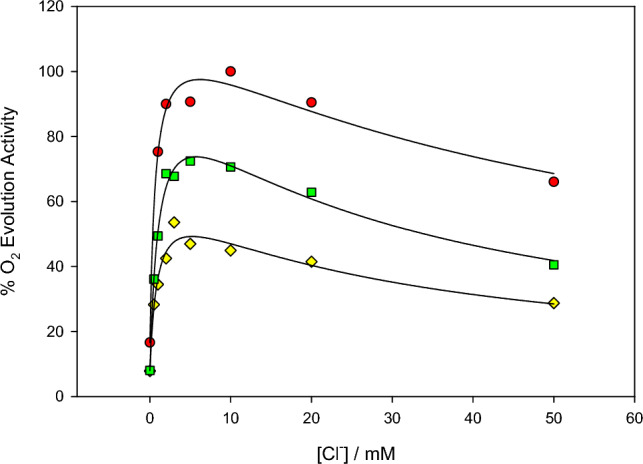
Dependence of O_2_ evolution activity on Cl^−^ concentration in the presence of various concentrations of Ca^2+^ at pH 5.5 using PSII lacking PsbP/PsbQ. Ca^2+^ concentrations were: red circles, 4.0 mM Ca^2+^; green squares, 1.0 mM Ca^2+^; and yellow diamonds, 0.5 mM Ca^2+^. Solid lines show direct fits to the data sets

The activation of O_2_ evolution by Cl^−^ at three different Ca^2+^ concentrations was studied at pH 5.5 (Fig. 4), which is below the optimal pH range of 6.0–6.5. In a previous pH dependence study, it was found that the activity became suppressed at high Cl^−^ concentrations (Baranov and Haddy 2017), which can be modeled with a second Cl^−^ site that is inhibitory. For each Ca^2+^ concentration, the activation by Cl^−^ was fitted to the modified Michaelis–Menten equation with substrate inhibition term (Eq. 2). The apparent K_M,app_(Cl^−^) was found to be from 0.7 to 1.1 mM for the three concentrations of Ca^2+^ tested and the apparent inhibition constant K_I,app_(Cl^−^) ranged from 29 to 57 mM (Table 3). For comparison, the same preparation at pH 6.3 showed a K_M,app_ of 1.9 ± 0.5 mM in the presence of sufficient Ca^2+^ (not shown).

**Table 3:** Apparent kinetic constants for Cl^−^ activation of PSII lacking PsbP/PsbQ at pH 5.5 at various Ca^2+^ concentrations. Values are from direct fits to the Michaelis–Menten equation with substrate inhibition term. Data correspond to the activation curves shown in Fig. 4. Errors given are standard errors of the fitted values

[Ca^2+^] / mM	K_M,app_(Cl^−^) / mM	V_max,app_	K_I,app_(Cl^−^) / mM
0.5	0.93 ± 0.28	56 ± 6%	29 ± 9
1.0	1.09 ± 0.16	91 ± 5%	30 ± 5
4.0	0.67 ± 0.20	99 ± 8%	57 ± 16

The data were analyzed according to the sequential bisubstrate activation model (Eq. 3), using the calculated values for K_M,app_(Cl^−^) which omitted the inhibitory effect at high Cl^−^ concentrations. The results are shown in Table 4.

**Table 4:** Kinetic constants obtained from the bisubstrate analysis of Ca^2+^ and Cl^−^ activation data, using Eq. 3 as described in Materials and Methods. One of the two values of K_S_ was determined from the relationship K_S_(Ca^2+^)K_M_(Cl^−^) = K_S_(Cl^−^)K_M_(Ca^2+^)

pH	6.3			5.5
Primary dependent variable ion	Ca^2+^ *	Cl^−^	Cl^−^ *	Cl^−^
K_S_(Cl^−^)/mM	11 ± 13	2.8 ± 1.6	7.5 ± 2.5	1.6 ± 0.7
K_M_(Cl^−^)/mM	2.4 ± 0.2	1.6 ± 0.2	2.1 ± 0.5	0.61 ± 0.10
K_S_(Ca^2+^)/mM	0.44 ± 0.17	0.26 ± 0.11	0.90 ± 0.30	1.1 ± 0.2
K_M_(Ca^2+^)/mM	0.10 ± 0.12	0.14 ± 0.06	0.25 ± 0.05	0.42 ± 0.19

The * indicates the inclusion of a Ca^2+^ depletion step

### Summary of kinetics data

The results of all four Ca^2+^ and Cl^−^ bisubstrate activation studies, including three at pH 6.3 and one at pH 5.5, are summarized in Table 4. For each set of data, the data were analyzed according to Eq. 3, leading to values for four kinetic constants (K_M_s and K_S_s); only three constants are independent, with the fourth calculated based on the symmetrical nature of the random sequential binding model. Although the model corresponding to random sequential binding was used to calculate values, the results indicate that the actual mechanism is not completely random, as described below.

In general, values found at pH 6.3 for the K_M_ and K_S_ values were consistent with previous studies in which only one ion was studied. Values for the chloride Michaelis constant, K_M_(Cl^−^), were in the range 1.6 to 2.4 mM, and the dissociation constant for Cl^−^, K_S_(Cl^−^), was found to be 2- to 4-fold higher in the range 2.8 to 11 mM. For calcium, values for K_M_(Ca^2+^) were in the range 0.10 to 0.25 mM, while the values for K_S_(Ca^2+^) were 2- to 4-fold higher in the range 0.26 to 0.90 mM. For most values, the error was between 8 and 40%, although two had errors above 100%. Although the constants show variation between the three NaCl-washed PSII preparations (which is expected based on past experience), the trends within each set were the same. In particular, K_S_(Cl^−^) > K_S_(Ca^2+^) and K_M_(Cl^−^) > K_M_(Ca^2+^), with no overlap of errors for five of the six pairs compared. This indicates an overall greater affinity of PSII for Ca^2+^ than for Cl^−^ by about an order of magnitude. It was also found that for each ion the Michaelis constant was less than the dissociation constant (i.e., K_M_(Cl^−^) < K_S_(Cl^−^) and K_M_(Ca^2+^) < K_S_(Ca^2+^)) for all three data sets, with no overlap of errors for three of the six pair of values compared.

At pH 5.5, both K_M_(Cl^−^) and K_S_(Cl^−^) were found to be lower than those constants at pH 6.3, with values of 1.6 mM and 0.6 mM, respectively. At the same time the values for K_M_(Ca^2+^) and K_S_(Ca^2+^) were higher than at pH 6.3, with values of 1.1 mM and 0.4 mM, respectively. Thus the overall affinity for Cl^−^ was higher and that for Ca^2+^ was lower, which can be considered consistent with the greater protonation environment at the lower pH. The pattern in which K_M_ < K_S_ still held for each activator at pH 5.5.

The trend in which K_M_ < K_S_ for both Ca^2+^ and Cl^−^ substrate activators of oxygen evolution indicates that the binding of the first ion promoted binding /activation by the second one for each binding path (Bisswanger 2017). This differs from a completely random binding mechanism, for which it is expected that K_M_^A^ = K_S_^A^ and K_M_^B^ = K_S_^B^. It also contrasts with a mechanism that favors ordered sequential binding with A binding first, for which it is expected that K_M_^A^ > K_S_^A^ and K_M_^B^ < K_S_^B^.

A second indicator of the nature of the bisubstrate activation by Ca^2+^ and Cl^−^ is found in the trends in apparent K_M_ and V_max_ values for activation by one ion when the other is held constant (Tables 1 and 2). For a completely random sequential model, simulations show that as fixed [B] increases both K_M,app_^A^ and V_max,app_^A^ increase and likewise as fixed [A] increases both K_M,app_^B^ and V_max,app_^B^ increase (Marangoni 2003). However for a strictly ordered sequential model (in which only one path is present), simulations show that as fixed [B] increases K_M,app_^A^ decreases and V_max,app_^A^ increases, while as fixed [A] increases, K_M,app_^B^ decreases at lower concentrations than seen for the other substrate and V_max,app_^B^ stays constant. (See Zheng et al*.* for an example of these two cases in histone methyltransferases (Zheng et al. 2021).)

In the present study, both sets of Cl^−^ activation data showed decreasing values of K_M,app_ and increasing values of V_max,app_ as the fixed Ca^2+^ concentration increased (Table 2), which is indicative of sequential binding with Cl^−^ as the first (A) substrate. For activation by Ca^2+^ at fixed Cl^−^ concentrations (Table 1), although a trend in K_M,app_ is not clear, V_max,app_ increased with Cl^−^ concentration. This is suggestive of sequential binding with Ca^2+^ as the first substrate. Neither Ca^2+^ nor Cl^−^ showed an increasing trend in K_M,app_ values expected for completely random binding; and neither showed constant V_max,app_ values expected if one binding path were disfavored in ordered binding. Rather the best interpretation is that both binding paths are favored and the binding of either Ca^2+^ or Cl^−^ first promotes the binding of the other in the activation of oxygen evolution.

### EPR spectroscopy of tyrosine radicals

The combined effects of Ca^2+^ and Cl^−^ on the redox active tyrosine radicals, Y_D_• and Y_Z_•, was examined using EPR spectroscopy. Both tyrosine residues transfer electrons to the donor side of PSII under illumination, however in active PSII Y_D_• is relatively dark-stable, decaying over a period of hours, whereas Y_Z_• disappears within milliseconds after formation as it picks up an electron from the Mn_4_CaO_5_ cluster. If electron transfer from the Mn_4_CaO_5_ cluster to Y_Z_• is inhibited, Y_Z_• can be trapped before it decays by backreaction with acceptor side electrons.

**Fig. 5:**
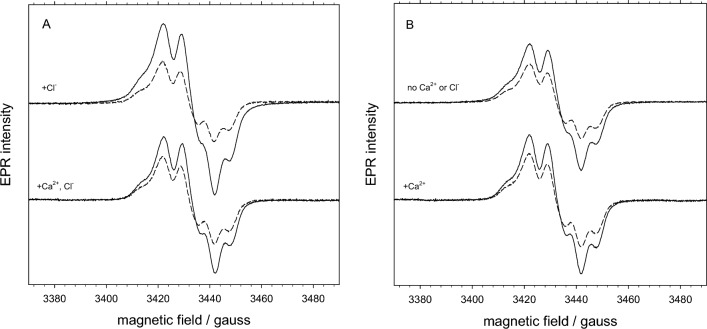
EPR spectra of tyrosine radicals in dark-adapted (dashed line) and illuminated (solid line) PSII lacking PsbP/PsbQ in the presence of: A bottom, 5 mM Ca^2+^ and 25 mM Cl^−^ (sample 1); A top, 25 mM Cl^−^ (sample 2); B bottom, 5 mM Ca^2+^ (sample 3); and B top, no Cl^−^ or Ca^2+^ (sample 4). Samples without Ca^2+^ were prepared with 1 mM EDTA to ensure absence of Ca^2+^, as described in Materials and Methods. EPR spectra were taken at 77 K using 1 mW power and 3 G modulation amplitude

In this experiment, PSII lacking PsbP/PsbQ was prepared using four different conditions in which buffer contained: 1, both 25 mM Cl^−^ and 5 mM Ca^2+^ (control); 2, 25 mM Cl^−^, but no Ca^2+^; 3, 5 mM Ca^2+^, but no Cl^−^; 4, neither Ca^2+^ nor Cl^−^. EDTA was added to facilitate Ca^2+^ removal for those samples (2 and 4) without Ca^2+^. The EPR spectrum of tyrosine radicals in the dark-adapted sample was compared with the spectrum after illumination for 30 s at 0 °C (Fig. 5), assuming that the dark-adapted sample represented complete formation of Y_D_•. A small amount of a simple isotropic radical signal was also induced by illumination, as can be seen by the relative increase in the second hyperfine peak from the left. This radical, which had a width of about 9 G, was found to contribute 10–20% of the overall signal height, so a correction was introduced to account for its contribution (see Materials and Methods).

The amount of Y_Z_• present after illumination was found to be 91% in the presence of Cl^−^ but no Ca^2+^ (sample 2, -Ca^2+^/ + Cl^−^) and between 39 and 50% in the other three samples (Table 5). Under ideal conditions, the control sample with sufficient Ca^2+^ and Cl^−^ (sample 1, + Ca^2+^/ + Cl^−^) would be expected to show little increase in tyrosine radical signal after illumination because of rapid reduction of Y_Z_•. While some additional Tyr radical was induced by illumination in all samples, it was evident that much more light-induced Tyr radical was produced in the absence of Ca^2+^ and presence of Cl^−^ (sample 2, -Ca^2+^/ + Cl^−^), compared to the other conditions. This indicates that Y_Z_• was unable to be reduced by the Mn cluster, as expected because without Ca^2+^ bound to the Mn cluster it cannot function in electron transfer. What is interesting is that in the absence of both Ca^2+^ and Cl^−^ (sample 4, -Ca^2+^/-Cl^−^), there was only a small increase in Y_Z_• signal over the control sample that contained sufficient amounts of both Ca^2+^ and Cl^−^ ions. This indicates that *there was a requirement for the presence of Cl*^*−*^* to observe the slow-decaying Y*_*Z*_*• signal*. The sample containing Ca^2+^ but no Cl^−^ (sample 3, + Ca^2+^/-Cl^−^), which showed slightly less radical than the control sample, is consistent with these observations; the presence of Ca^2+^ would promote reduction of Y_Z_• and the absence of Cl^−^ would prevent observation of Y_Z_• in the few centers where Ca^2+^ was absent. The requirement for Cl^−^ to observe the induced Y_Z_• radical suggests that it is either required for its formation or stabilizes it after formation.

**Table 5:** Y_Z_• radical induced by illumination as observed by EPR spectroscopy in PSII lacking PsbP/PsbQ in the presence and absence of Ca^2+^ and Cl^−^. Samples, which correspond to those in Fig. 5, were prepared and EPR signals were measured in dark-adapted and illuminated states as given in Materials and Methods. Signals were quantified using peak-to-trough heights (h) and the ratios of illuminated to dark-adapted heights are given in column 3. The calculated amount of Y_Z_• after a correction for a narrow light-induced radical is given in column 4; the correction introduced error of ± 2%

Sample	Buffer conditions		h (illum)/ h (dark)	% Y_Z_• (after correction)
	5 mM Ca^2+^	25 mM Cl^−^		
1	+	+	1.6	43
2	−	+	2.1	91
3	+	−	1.5	39
4	−	−	1.7	50

The presence of some Y_Z_• signal in all of the samples, including the control, is probably an indication of the presence of some nonfunctional O_2_ evolving centers combined with the presence of some Cl^−^ that carried over during treatment. Treatment conditions were kept mild, to balance maintaining the integrity of the sample with adequate removal of Ca^2+^ and/or Cl^−^. Experience in earlier versions of this experiment indicated how easily samples were impaired, resulting in weak signal formation and little difference between the samples, as well as increased interference from the narrow radical signal. NaCl-washing to remove the extrinsic PsbP and PsbQ subunits is known to result in reduced activity, probably because of damage to the OEC centers.

### EPR spectroscopy of OEC signals at 10 K

In another EPR experiment, samples of PSII lacking PsbP/PsbQ were prepared similarly to those described above, with and without Ca^2+^ or Cl^−^, except at a higher concentration of PSII for examination of additional signals from the OEC. Conditions were established to examine three signals at 10 K: the dark-stable multiline signal associated with the inhibited S_2_ (S_2_’) state, the normal S_2_-state multiline signal, and the S_2_Y_Z_• signal due to inhibition of electron transfer from the Mn cluster to Y_Z_•.

**Fig. 6:**
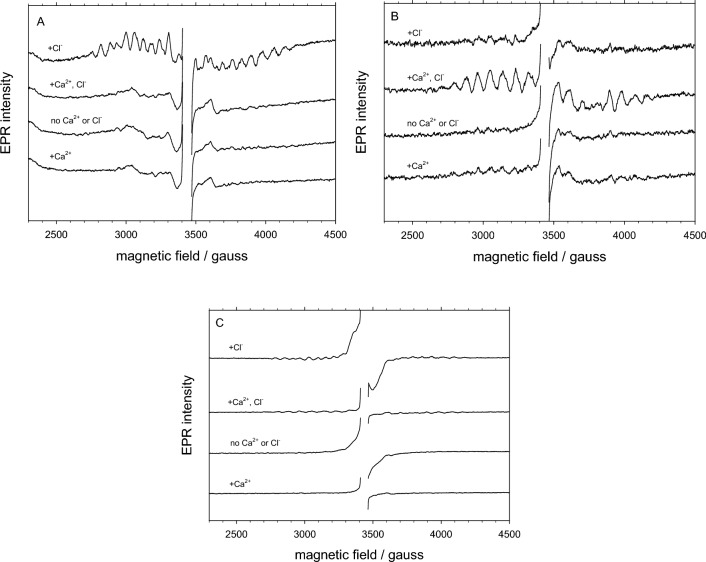
EPR spectra of PSII lacking PsbP/PsbQ that had been dark-adapted (**A)**, followed by illumination at 195 K (**B)**, and finally by illumination at 273 K (**C)**. Panels B and C show difference spectra resulting from subtraction of the dark-adapted spectra of Panel A. Samples were prepared as described in Materials and Methods with: top, 25 mM Cl^−^; second, 6 mM Ca^2+^ and 25 mM Cl^−^; third, no Cl^−^ or Ca^2+^; and bottom, 6 mM Ca^2+^. Samples also contained 0.1 mM EDTA. EPR spectra were taken at 10 K using 20 mW power and 18 G modulation amplitude. For comparison, the intensity scales in Panels B and C are 0.63 and 5.2 relative to that of Panel A. (The top spectrum in panel A was published previously in (Haddy and Ore 2010).)

PSII samples were first illuminated at room temperature and dark-adapted on ice. In active PSII, such as that containing both Ca^2+^ and Cl^−^, this poises the centers in the S_1_ state. However, in Ca^2+^-depleted PSII, this results in the dark-stable multiline signal which represents an inhibited S_2_ state (S_2_’) that has been produced by dissociation of Ca^2+^ in the S_3_ state, followed by single-electron reduction to the S_2_’ state. (For PSII prepared in the absence of Ca^2+^ in the dark, Ca^2+^ is not lost from the Mn cluster until illumination.) In the four samples examined (Fig. 6, panel A), only the Ca^2+^-depleted sample containing Cl^−^ (-Ca^2+^/ + Cl^−^) showed the dark-stable multiline signal, whereas the sample with neither Ca^2+^ nor Cl^−^ (-Ca^2+^/-Cl^−^) showed no such signal.

Next, samples were illuminated at 195 K, which results in formation of the normal S_2_ state multiline signal in active samples (Fig. 6, panel B). As expected, this signal was observed in only the sample containing both Ca^2+^ and Cl^−^ (+ Ca^2+^/ + Cl^−^). A very weak S_2_ state *g* = 4.1 signal was also observed in the same sample (not shown), but not in any of the others.

The final illumination at 273 K (Fig. 6, panel C) produced the S_2_Y_Z_• signal only in the Ca^2+^-depleted sample containing Cl^−^ (-Ca^2+^/ + Cl^−^), which was the same sample that showed the dark-stable multiline signal; the latter signal can still be seen in the baseline of the spectrum. The illumination protocol that produced the S_2_Y_Z_• signal was very similar to that used in the previous experiment to observe the Y_Z_• signal at 77 K, except with the intermediate illumination at 195 K; the two signals originate from the same radical, but at 10 K the interaction with the S_2_’ state Mn cluster results in broadening of the signal. (A small amount of broad radical signal can be seen in the sample with no Ca^2+^ or Cl^−^ (-Ca^2+^/-Cl^−^), which can be explained by the presence of a trace amount of Cl^−^.)

## Discussion

### *Bisubstrate enzyme kinetics of activation by Ca*^*2*+^*and Cl*^*−*^

In this study, the activation of oxygen evolution by Ca^2+^ and Cl^−^ ions in PSII lacking PsbP and PsbQ was analyzed in terms of bisubstrate enzyme kinetics. Using a sequential binding model in which either ion can bind first, four kinetic constants (K_M_s and K_S_s) were obtained (Table 4). Although not substrates, modeling ion activators as substrates in kinetics studies has been well established in the photosystem II literature. The catalytic cycle of oxygen evolution is highly complex, but the application of relatively simple enzyme kinetics models can work in practice because key steps dominate the observed kinetics.

For chloride, the dissociation constant for first step binding, K_S_(Cl^−^), was found to be 2.8 to 11 mM and the Michaelis constant for second step binding, K_M_(Cl^−^), was 1.6 to 2.4 mM. These values are in the ranges found in previous studies for each ion activator. K_M_ for Cl^−^ activation in PSII lacking PsbP and PsbQ has been reported to be 1 to 3 mM (Haddy et al. 2017; Kelley and Izawa 1978; Miyao and Murata 1985; Wincencjusz et al. 1998). These studies, as well as that reported here, support the presence of a single type of activating Cl^−^. Studies by Lindberg and Andreasson, using higher plant PSII with the extrinsic subunits present, characterized high affinity (K_d_ = 20 μM) and low affinity (K_d_ = 0.5 mM) forms of the Cl^−^ binding site that were associated with an interconversion in response to pretreatment conditions (Lindberg and Andréasson 1996).

For calcium, the dissociation constant, K_S_(Ca^2+^), was found to be 0.26 to 0.90 mM and the Michaelis constant, K_M_(Ca^2+^), was found to be 0.10 to 0.25 mM. Values found in previous studies generally represent two affinities for Ca^2+^, one in the micromolar range and the other in the low millimolar range (Miqyass et al. 2007; Yocum 2008). In particular, for PSII depleted of the PsbP and PsbQ subunits by NaCl-washing, activation studies found K_M_ values of 55 μM and 2.2 mM (Han and Katoh 1995) and binding studies using ^45^Ca^2+^ found K_d_ values of 26 μM and 0.5 mM (Ädelroth et al. 1995). In contrast to these earlier studies, in the study carried out here no biphasic dependence of activation was observed, as Ca^2+^ activation curves at fixed Cl^−^ concentrations were fitted well with a single K_M,app_ for Ca^2+^ (given a small amount of initial activity, V_0_, attributed to bound Ca^2+^). This could be because the Ca^2+^ depletion treatment produced a homogeneous preparation. Values determined here for K_M_(Ca^2+^) and K_S_(Ca^2+^), which were between 0.10 and 0.90 mM, are close to the low affinity values determined in previous studies.

A bisubstrate analysis of Ca^2+^ and Cl^−^ activation was also carried out at pH 5.5. The lower pH is of interest because in a previous study it was found to promote Ca^2+^ loss at elevated Cl^−^ concentrations (Haddy and Ore 2010). In the present study, the K_M_ and K_S_ values for Ca^2+^ at pH 5.5 were found to be higher than at pH 6.3, consistent with this observation, while the K_M_ and K_S_ values for Cl^−^ were found to be lower. Also at pH 5.5, a marked inhibitory effect was observed as Cl^−^ concentrations increased. Fits to the activation curves at fixed Ca^2+^ concentrations gave values of apparent Michaelis constants K_M,app_(Cl^−^) around 1 mM and apparent Cl^−^ inhibition constants K_I,app_(Cl^−^) of 29 to 56 mM. In a previous study of pH and chloride dependence of PSII lacking the PsbP and PsbQ subunits (Baranov and Haddy 2017), the intrinsic pH-independent dissociation constants for Cl^−^ activation and Cl^−^ inhibition were found to be 0.9 mM and 64 mM, respectively, similar to the apparent constants found here.

As described in Results, the best interpretation of the bisubstrate kinetic data is that either Ca^2+^ or Cl^−^ can bind first and that the first bound ion promotes binding of the second. This is based on the observation that for each ion K_S_ (representing first-step binding) was found to be two- to fourfold greater than K_M_ (representing second-step binding). This interpretation is also supported by the trends in K_M,app_ and V_max,app_ for each ion at various fixed concentrations of the other ion. How this kinetic mechanism might occur, given the complexity of the water oxidation cycle in photosystem II, may be gleaned by considering the known characteristics of Ca^2+^ and Cl^−^ during activation of oxygen evolution.

Based on previous studies of the association of each ion during the S-states, it is reasonable to expect that activating Ca^2+^ binds to the OEC prior to Cl^−^. Calcium is required for each S-state transition because it must be bound at the Mn_4_CaO_5_ cluster for electron transfer to Tyr Z. Chloride is usually present at each S-state transition, but it is possible to achieve the S_2_ state in its absence while advancement to the S_3_ state is blocked in the absence of Cl^−^ (Theg et al. 1984; Itoh et al. 1984). In addition, by directly detecting Mn oxidation state changes using flash-induced UV absorbance, it was shown that Cl^−^ is required only on the S_2_-to-S_3_ and S_3_-to-S_0_ transitions (Wincencjusz et al. 1997). It is noteworthy that the characteristics of the S_2_ state are altered in the absence of Cl^−^: the formation of the S_2_-state multiline signal at *g* = 2 is inhibited while the S_2_-state *g* = 4.1 signal can still form (Ono et al. 1987; Haddy 2007). Chloride at the Cl−1 site has a role in proton translocation involving the D1-Asp61 residue. Early evidence indicating this was provided by mutagenesis studies (Dilbeck et al. 2012; Debus 2014). More recently, computational modeling studies have supported the formation of an inhibitory salt-bridge between D1-Asp61 and D2-Lys317 in the absence of chloride (Pokhrel et al. 2011; Rivalta et al. 2011) and a role for the Cl−1 chloride in controlling the release of a proton from water W1 to D1-Asp61 (Yang et al. 2021; Saito et al. 2020). Given the importance of its role in proton translocation, chloride is probably necessary for optimal function in the early S-states, even if not strictly required for each transition.

While the above observations indicate that Ca^2+^ is bound during the S-state cycle before Cl^−^ is necessarily required, the observed kinetics probably reflect a narrower range of events during the cycle. The affinity of the OEC for both Ca^2+^ and Cl^−^ decreases in the higher S-states. For Cl^−^, K_d_ was estimated to be 80 μM in S_1_, 1.0 mM in S_2_, and 130 mM in S_3_ at pH 6.0 (Wincencjusz et al. 1998). For Ca^2+^, the lower affinity in the higher S-states is shown by an increase in dissociation rates (Ädelroth et al. 1995). It is thought that during Ca^2+^ depletion treatments, Ca^2+^ is released from its binding site at the OEC during the S_2_ or S_3_ state (Miqyass et al. 2007), thus Ca^2+^ depletion procedures often include an illumination step in addition to the use of chelators. Under normal conditions, the release of Ca^2+^ and Cl^−^ is prevented by the presence of the extrinsic subunits. Given these considerations, the kinetics are likely to reflect rate-limiting steps of the catalytic cycle in which both chloride and calcium ions can undergo local association-dissociation events. This would be consistent with the observation that binding paths are available in which either ion can bind first. The ability of each ion to promote the binding of the other is probably due to structural effects through the Mn_4_CaO_5_ cluster and its surrounding environment.

The calcium ion of the OEC is not closely linked to either of the two chloride ions (Cl−1 and Cl−2), thus the mode of mutual support provided by one ion for the other must take place indirectly. The water channels and coordinating ligands at the OEC show possible routes of interaction through hydrogen bonding. As part of the Mn_4_CaO_5_ cluster, the Ca^2+^ ion is coordinated by D1-Asp170 and linked to the nearby Tyr Z residue (D1-Tyr161) through several hydrogen bonded water molecules, including W3 and W4 (Umena et al. 2011; Kawakami et al. 2011; Kern et al. 2018). The Cl^−^ ions are each coordinated by amide N atoms from residues with sidechains coordinated to two Mn atoms of the Mn_4_CaO_5_ cluster: Cl−1 by D1-Glu333, which coordinates Mn3 and Mn4, and Cl−2 by CP43-Glu354, which coordinates Mn2 and Mn3. Three major water channels, designated Cl1, O1 and O4, lead to the solvent-exposed surface of PSII (Suga et al. 2019; Kern et al. 2018; Hussein et al. 2021). The chloride ion at the Cl−1 site is associated with the Cl1 channel, while the chloride ion at the Cl−2 site is associate with a smaller network of water molecules. A comparison of water mobility finds that those in the Cl1 and O4 channels are less mobile than in the O1 channel (Ibrahim et al. 2020), which supports a role for Cl1 in proton transfer, complementing other studies of the role of nearby residues such as D1-Asp61. The Cl1 and the O1 channels appear to be linked through several water molecules, notable W2, W3, W4 and others. This network of waters could provide a route through which Ca^2+^ and Cl^−^ may influence each other via hydrogen bonding.

The identity of the chloride responsible for activation of O_2_ evolution is not revealed by this study, but structural characteristics are consistent with its identity as the Cl−1 chloride. As already noted, this and previous studies of higher plant PSII lacking the PsbP and PsbQ subunits support the activation of O_2_ evolution by a single type of Cl^−^ ion. The ligation of Cl^−^ at the Cl−1 site includes the Lys-317 sidechain amine, indicating that it is bound with higher affinity than the Cl^−^ at Cl−2. However, a more important factor in the exchangeability of Cl^−^ may be access to the external medium. The Cl1 channel appears to provide the Cl−1 chloride with greater access to the surface of PSII than the small water network of the Cl−2 chloride. Thus, the Cl−1 chloride may have the lability required to display the type of exchangeable kinetics seen in this and previous studies.

### EPR spectroscopy of the Tyr Z radical (YZ•)

Using EPR spectroscopy, we observed the tyrosine radical signals at 77 K to determine the amount of light-induced reduced Y_Z_• in PSII lacking PsbP and PsbQ after various Ca^2+^ and/or Cl^−^ depletion treatments (Fig. 5, Table 5). We found that the 77-K slow-decaying Y_Z_• signal due to Ca^2+^ depletion was only observed in the presence of Cl^−^. Using low temperature EPR at 10 K, we also observed the S_2_Y_Z_• signal induced by Ca^2+^ depletion using similarly prepared samples (Fig. 6), also with the observation that Cl^−^ was required for its formation.

The slow-decaying Y_Z_• signal induced by Ca^2+^ depletion is identified with the S_2_Y_Z_• signal characterized previously in Ca^2+^-depleted PSII (Boussac et al. 1989; Ono and Inoue 1990; Sivaraja et al. 1989; Hallahan et al. 1992). The signal has been demonstrated to be due to coupling of Y_Z_• with the *S* = 1/2 spin state of the S_2_ state Mn cluster (Zahariou et al. 2021), which causes broadening to a width of 135 to 165 G when observed at liquid helium temperatures of 5–20 K. Similar signals have been observed in fluoride-treated PSII (Baumgarten et al. 1990; DeRose et al. 1995) and acetate-treated PSII (MacLachlan and Nugent 1993; Szalai and Brudvig 1996; Tang et al. 1996; Lakshmi et al. 1998), which also show impaired electron transfer to Tyr Z. At 77 K Y_Z_• is uncoupled from the Mn cluster and has a very similar appearance to Y_D_•, as has been demonstrated in the acetate-induced S_2_Y_Z_• signal (Szalai et al. 1998). Our observation of the signal at 77 K, where the hyperfine pattern is clear, ensures its identification as tyrosine and eliminates possible confusion with other organic radicals. The Y_Z_• signal at 77 K in the Ca^2+^-depleted PSII with sufficient Cl^−^ (−Ca^2+^/ + Cl^−^) correlated well with the S_2_Y_Z_• signal at 10 K, while the absence of the S_2_Y_Z_• signal in the other three samples suggests that the small amount of Tyr radical observed at 77 K did not involve interaction with the Mn cluster.

Two other signals associated with the nearby Mn cluster of the OEC were also observed at 10 K: the S_2_-state multiline signal of active PSII and the dark-stable multiline signal associated with Ca^2+^-depleted PSII. These help to provide insight into the electron transfer processes in each sample and the reason for the requirement for Cl^−^ to observe the slow-decaying Y_Z_• or the S_2_Y_Z_• signals in Ca^2+^-depleted PSII. During the dark-adaptation period that follows brief room-temp illumination, active PSII centers in the presence of both Ca^2+^ and Cl^−^ become coordinated in the S_1_ state. With sufficient Ca^2+^ and Cl^−^, this control sample (+ Ca^2+^/ + Cl^−^) showed the normal S_2_-state multiline signal after illumination at 195 K and it showed neither of the signals associated with Ca^2+^ depletion. The Ca^2+^-depleted PSII sample containing Cl^−^ (−Ca^2+^/ + Cl^−^) is noteworthy in that it was the only sample to show the dark-stable multiline signal after the illumination/dark-adaptation step. In these treatment conditions, Ca^2+^ is thought to be lost in the S_3_ state during illumination, after which the impaired Mn clusters reduce to the dark-stable S_2_’ state. (Samples were prepared by buffer washes in the dark, which can effectively remove Cl^−^, but a significant fraction of Ca^2+^ remains bound to the OEC.) In the same sample, the presence of the dark-stable multiline signal correlates with the observation of the S_2_Y_Z_• signal after the final illumination at 273 K. In the Ca^2+^-depleted PSII without Cl^−^ ﻿(-Ca^2+^/-Cl^−^), the OEC centers were unable to advance to S_3_ in the absence of Cl^−^, thus any Ca^2+^ present did not dissociate and the dark-stable multiline signal did not appear. In this sample, the S_2_Y_Z_• signal also did not form because the S_2_’ state was not present, whereas for the −Ca^2+^/ + Cl^−^ sample, the S_2_’ state was already present at the time of the final illumination at 273 K. Finally, the PSII sample with sufficient Ca^2+^ but no Cl^−^ (+ Ca^2+^/-Cl^−^) also showed none of the EPR signals. It was unable to advance to the normal S_2_ state in the absence of Cl^−^ (and also did not show the *g* = 4.1 signal). It is noteworthy that it also did not enter an EPR-silent S_2_ state that could interact with Y_Z_• to produce a modified S_2_Y_Z_• signal.

These results indicate that the requirement for Cl^−^ in the formation of the slow-decaying Y_Z_• or S_2_Y_Z_• signal is primarily related to the requirement for Cl^−^ to attain the S_3_ state, from which Ca^2+^ is released. The reason for this may be related to the role of chloride in proton transfer during water oxidation. The established pattern of proton release to the external medium for S-state transitions from S_i_ = S_0_ through S_4_ is 1:0:1:2 (Renger 2012). However, in the extended scheme of the S-state cycle described by Dau and Haumann (Dau and Haumann 2008, 2007), proton transfer from the Mn_4_CaO_5_ cluster precedes Mn oxidation by electron transfer to Tyr Z in each of the S_i_ states; thus S_i_^+^  → S_i_^n^, where + denotes positively charged and n denotes neutral. In this scheme, the observed classical S_0_ and S_1_ states correspond to the neutral S_0_^n^ and S_1_^n^ states (after proton transfer), whereas the observed classical S_2_ and S_3_ states correspond to the S_2_^+^ and S_3_^+^ states (before proton transfer). Studies using time-resolved photothermal beam deflection (PBD) have resolved changes that support this model. Among other findings, it was shown that proton release must precede electron transfer from the Mn cluster to reduced Y_Z_• during the S_2_-to-S_3_ transition (Klauss et al. 2012a, 2015, 2012b). Given that in the extended S-state model the S_2_ state would correspond to the S_2_^+^ state before proton transfer, chloride would have an important role in facilitating the proton transfer that precedes formation of the S_3_ state. This still leaves open the question of why Y_Z_• in the S_2_Y_Z_• state is relatively stable to backreaction with the acceptor side. In their study of time-resolved details of the S_2_-to-S_3_ transition, Klauss and coworkers (Klauss et al. 2012b) described nanosecond time frame volume changes due to nuclear rearrangements in the S_2_ state that must precede proton removal from the Mn cluster and electron transfer to Tyr Z; they discuss these changes in the context of stabilizing the oxidized Y_Z_• to prevent backreaction with Q_A_^−^. Given the role of Cl^−^ in proton transfer and its potential interaction via the cluster of water molecules near Tyr Z, it is possible that chloride also has a role in the stabilization of Y_Z_•.

### Concluding remarks

Using a bisubstrate enzyme kinetics approach, we have found that activation of oxygen evolution by Ca^2+^ and Cl^−^ shows kinetics consistent with a sequential binding model in which either ion can bind first. At the same time, the relative kinetic constants indicate that each promotes the activation of oxygen evolution by the other. This suggests a low-level of interdependence that may correspond to coupling through a hydrogen bond network. Study of the Y_Z_• and S_2_Y_Z_• EPR signals in Ca^2+^-depleted PSII also suggests an important role for Cl^−^ in the formation of the S_2_’ state and in stabilizing the Y_Z_• radical, perhaps as a part of its role in proton transfer that precedes electron transfer from the Mn_4_CaO_5_ cluster. Both of these effects could take place through the water network around the Mn_4_CaO_5_ complex. Structural studies offer details about how this could occur. The chloride ion at the Cl−1 site is in close proximity with water molecule W21 which is linked to the Ca^2+^ ion through water molecules that include W2, W3, W22, and W23 (Kern et al. 2018; Suga et al. 2019). The Tyr Z residue is also a part of this network, with interaction with Ca^2+^ through waters W3, W4, and W25. Hydrogen bonding distances between the ions and nearby water molecules are seen to shift on a femtosecond time scale during the course of the S_2_-to-S_3_ transition (Kern et al. 2018; Hussein et al. 2021), which is a key step leading up to the formation of oxygen. Interactions such as these between the sites moderating the proton transfer and Mn oxidation must take place throughout the S-state cycle in a dynamic coordination of catalysis.

## Data Availability

Data supporting the findings of this study are included in the article and are available upon reasonable request.
